# A Case Study on Recycling Industrial Wastewater with Nanofiltration Membrane Separation Technology

**DOI:** 10.3390/membranes14120266

**Published:** 2024-12-09

**Authors:** Yiqiang Deng, Xiaoqian Bai, Jialong Lin, Linyan Yang, Yuqing Lin, Mingyue Lin, Guangli Xiu

**Affiliations:** 1 State Environmental Protection Key Laboratory of Environmental Risk Assessment and Control on Chemical Process, East China University of Science and Technology, Shanghai 200237, China; yiqiang_deng@pmmail.eternal-group.com.cn (Y.D.); y83230070@mail.ecust.edu.cn (X.B.); y30221123@mail.ecust.edu.cn (J.L.); lyyang@ecust.edu.cn (L.Y.); linyuqing@ecust.edu.cn (Y.L.); 2 Shanghai Environmental Protection Key Laboratory on Environmental Standard and Risk Management of Chemical Pollutants, School of Resources and Environmental Engineering, East China University of Science and Technology, Shanghai 200237, China; 3 Shanghai Institute of Pollution Control and Ecological Security, Shanghai 200092, China

**Keywords:** nanofiltration membranes, water reuse, circulating cooling water

## Abstract

As pressure on water resources intensifies and stringent regulations for groundwater and surface water are enacted, wastewater recycling has emerged as a key research objective for many enterprises. In this study, based on the actual wastewater discharged from Eternal Electronic (Suzhou, China) Co., Ltd., the performance differences in different membrane materials in treating this wastewater were analyzed and compared. The NF90 membrane was ultimately selected as the most suitable choice for treating this wastewater with optimal operating conditions of 150 psi, 25 °C, and 1 L/min, respectively. All the indices of wastewater treatment can satisfy the standard of circulating cooling water. In addition, this work is closely combined with the practical applications by using an HCl solution (pH = 3–4) to clean the fouled membranes, thus effectively solving the membrane fouling in the treatment process. This approach not only satisfies the environmental management requirements of enterprises but also provides valuable insights for similar wastewater treatment projects.

## Simple Summary

In this study, the performance of different membrane materials for treating wastewater discharged by Eternal Electronic (Suzhou, China) Co., Ltd. was analyzed. NF90 membrane was selected to have the best treatment effect under the conditions of 150 psi, 25 °C, and 1 L/min, and the treated wastewater reached the standard of circulating cooling water. The problem of membrane contamination was effectively solved by cleaning with an HCl solution. This study aims to provide enterprises with environmental solutions to meet environmental management requirements and to provide a valuable reference for similar wastewater treatment projects. This method is helpful in relieving the pressure on water resources and has positive significance for the protection and sustainable utilization of water resources in society.

## 1. Introduction

With the introduction of strict control systems for groundwater and surface water, wastewater recycling has become a new goal for major companies to study. At present, total wastewater from boilers, cooling pools, water softening units, etc. is widely recognized internationally and domestically as an alternative water source that can be implemented. For power plants that have traditionally used groundwater and surface water as a source of boiler make-up water, changing the source of water production is imperative. Therefore, the development of zero-discharge wastewater technology as a source of boiler make-up water has important practical significance and broad application prospects.

The 2945 cubic meters of industrial wastewater generated by an electronic company located in southeast China mainly originate from boilers, cooling basins, water softeners, and laboratories. This wastewater, which was collected in 2022, contains complex components such as salts, suspended solids, organics, oils and greases, hardness ions, and possible chemical reagents, which need to be treated by processes such as coagulation and sedimentation, air flotation, chemical/ion exchange, biological and advanced treatments (e.g., advanced oxidation, membrane separation), etc., to ensure that the wastewater meets national or local discharge standards.

Feedwater with poor quality not only leads to scaling in the boiler but also significantly reduces metal strength and leads to local deformation, expansion, and cracking [1]. The accumulation of salts and water hardness significantly heightens operational safety risks. Therefore, the use of high-purity feedwater is essential for maintaining stable and efficient boiler operations [2]. The main traditional methods used to treat cooling water are capacitive ionization [3], membrane distillation [4], reverse osmosis [5], ultrafiltration [6], nanofiltration [7], and so on.

The adoption of membrane technologies is seen as a critical global strategy to meet the challenges of global water resources [8]. Membrane technology plays an important role in several industries, one of which is the treatment of groundwater and salt water to remove heavy metal ions [9,10]. Among the various membrane technologies available for water purification, reverse osmosis (RO) remains the most widely adopted due to its simplicity and energy efficiency. However, it is still plagued by issues related to fouling and requires high-pressure operation [8,11]. The most typical reverse osmosis membrane is the XLE membrane, which has excellent anti-fouling properties and better cleanliness. XLE has a compact structure and a small effective aperture radius (0.30–0.31 nm), which is the main reason for its high retention rate. The size exclusion also plays an important role in the rejection rate [12].

However, apart from high rejection values for multivalent ions, nanofiltration (NF) membranes can maintain higher water permeability compared to RO membranes [13]. NF is a membrane filtration technology that separates ions from molecules by the specific properties of the membrane, based on the size and charge of the ions [14]. The MWCO of nanofiltration membranes ranges from 200 to 1000 Da, intermediate between ultrafiltration and reverse osmosis [15]. Among many nanofiltration membranes, the NF90 membrane can be used under very low operating pressure, and the rejection of magnesium sulfate can reach more than 97% (according to the manufacturer). The mean pore size of NF90 was reported to be 0.99 nm [16,17]. The NF270 membrane is effective in removing organic matter and reducing hardness in surface water and groundwater, while the XN45 membrane offers advantages such as higher filtration precision and increased water flux.

The problem of membrane contamination is almost inevitable in the membrane separation process such as nanofiltration and reverse osmosis, and, even under strict pretreatment measures, pollutants may still accumulate on the surface of the membrane, resulting in decreased membrane performance. Adding acid is a simple and effective way to prevent calcium carbonate from scaling. A common acid used to reduce the pH of feedwater is sulfuric acid (H_2_SO_4_) [18]. HCl is a better acid than H_2_SO_4_ because sulfate is generally less soluble than chloride. Adding a small amount of effective anti-scale agents, such as polyelectrolytes, polyphosphates, and organophosphorus compounds to change the properties of the solution is also a very effective method to control the scale formation of inorganic salts such as CaCO_3_ and CaSO_4_ formed on the surface of NF/RO membranes [19]. If the concentration of some potential sediments is too high, the use of scale inhibitors is not recommended, as they do not completely prevent precipitation at high ion concentrations.

Herein, this study focuses on NF and RO membranes in treating wastewater from boilers, cooling ponds, water softeners, and laboratories. We selected four types of membranes to evaluate their performance in specific industrial effluents. The goal of this study is to determine the membrane type that best suits the wastewater treatment needs of the enterprise and optimize its operating parameters to improve wastewater reuse efficiency, reduce treatment costs, and meet the environmental requirements of the enterprise. The purpose of this study is to provide practical solutions for the treatment of the same type of wastewater and to provide references for the design and operation of related projects.

## 2. Experimental

### 2.1. Material

In this work, phenolphthalein, ammonium chloride, and chrome Black T were obtained from Shanghai Macklin Biochemical Technology (Shanghai, China) Co., Ltd. Methyl orange, sodium carbonate anhydrous, EDTA disodium magnesium, and triethanolamine were purchased from Shanghai Aladdin Biochemical Technology (Shanghai, China) Co., Ltd. Ammonia, EDTA disodium dihydrate, sodium cyanide, ammonium acetate, N, N-Diethyl-1,4-phenylenediamine sulfate, and potassium dihydrogen phosphate were purchased from commercial supplier Sinopharm Chemical Reagent (Shanghai, China) Co., Ltd. The above drugs were purchased from Sinopharm Chemical Reagent (Shanghai, China) Co., Ltd., and all of the reagents were used as received without further purification.

In this study, four commercial membranes NF90, NF270, XN45, and XLE were selected for filtration experiments to evaluate their performance in the treatment of different industrial wastewater. These four kinds of membranes have their own characteristics, with different physical and chemical properties and application advantages. NF90 and NF270, as nanofiltration membranes, have high salt and organic removal rates while maintaining a certain amount of water production. They are suitable for the treatment of wastewater containing high salt and organic matter and can meet the treatment needs under different water quality conditions. Among them, the salt removal rate of the NF90 membrane is higher, which is suitable for the application of more stringent salt requirements. The NF270 membrane can retain certain minerals while removing organic matter and is more suitable for water sources that require partial softening. The XN45 membrane is suitable for the treatment of substances containing insoluble substances. As a reverse osmosis membrane, the XLE membrane has the characteristics of a high desalting rate and high water yield, stable structure, and easy cleaning. It is suitable for the treatment of wastewater with high salinity and high hardness, which can significantly improve the efficiency of wastewater reuse and reduce the treatment cost.

In summary, the selection of these four membranes fully considers their applicability and advantages in different industrial wastewater treatments, aiming to optimize the operating parameters of membrane technology by comparing their performance and providing a valuable reference for practical industrial applications.

All membrane materials in the experiment were purchased from RisingSun Membrane Technology Co., Ltd. These membranes are made of polyamide (PA). The size of the membrane used in the experiment is 42 cm^2^. The virgin membrane properties, including water flux, the interception molecular weight, and the rejection rate are shown in Table 1. The Table 1 data are from the above manufacturer.

Flat membrane components were used in the laboratory. The membranes were installed in special membrane housing and can be replaced and cleaned separately. The membrane was installed in parallel, multiple membrane components that are connected side by side, and the water flow was distributed to each component. The wastewater was put into the reservoir, and the membrane with an area of 42 cm^2^ was placed in the test tank.

### 2.2. Wastewater Used in the Experiment

The water samples in this experiment came from Eternal Electronic (Suzhou, China) Co., Ltd. The company mainly develops and produces photoresist dry membranes, solder-proof dry membranes, electronic special materials, and mechanical equipment manufacturing. The wastewater covered multiple sources, including storage tanks, quality control workshops, adhesive coating workshops, and soft water daily tanks. The sources and parts of wastewater are shown in Table 2. The preliminary test results are shown in Table 3. The following water quality data provide basic information for the further optimization of wastewater treatment.

### 2.3. Experimental Methods

The membrane separation system was adopted as the reuse treatment system. The effluent water quality satisfies the national standard for industrial water reuse in China 19923-2024 [20] for circulating cooling water quality standards (Table 4).

The specific experimental steps are the following:

(a) Firstly, an optimal membrane material for treating the wastewater was selected among the four membrane materials. Four membranes, NF90, NF270, XN45, and XLE, were used to conduct filtration experiments at the same time, with a running time of 4 h, a membrane system pressure of 100 psi, a flow rate of 1 L/min, a temperature of 25 °C, maintaining the pH of the raw water, and the volume of feedwater of 20 L. Samples were taken to test water flux and salt rejection every half an hour, and samples were collected after 4 h to determine the COD value and other relevant parameters of the treated wastewater. Based on the results of the experiments, one suitable membrane material was selected. The above experiments were conducted a total of four times, maintaining consistent experimental conditions and parameters throughout. The test results presented are the average values derived from these four repetitions. Specific data from repeated experiments, including original measurements, calculated averages, and standard deviations, will be presented in the Supplementary Material.
(b) Tests were conducted with reference to the Dow Membrane Technical Manual (2022) for operating parameters. An orthogonal testing method was used to analyze the effects of temperature, pressure, and flow rate on the membrane operating parameters.
(c) According to the optimized conditions, long-term experiments were carried out. At the same time, after 12 h of operation, the membranes were cleaned in different ways, using NaOH, clean water, and HCl, respectively, and the membranes were cleaned every 6 h. The cleaning method was as follows: remove the membranes, put them in the cleaning solution, shake the membranes at room temperature for 1 h, rinse them with tap water after removal, and then return them to the membrane device for further operation. We compared different cleaning methods for membrane fouling and selected the most suitable treatment for practical application.

### 2.4. Methods of Analysis and Characterization

The chemical oxygen demand (COD) was measured in strict accordance with the Chinese national standard (GB/T 11914-2022 [21]). The total hardness of the water was determined using the EDTA titration method based on the international standard (ISO 6059-1984 [22]). Total alkalinity was assessed through the acid titration method by the Chinese industry standard (SL 83-1994 [23]). The contents of total chlorine and total iron were determined by referencing the Chinese industry standards (HJ 585-2010 [24], HJ 345-2007 [25]). Scanning electron microscopy (SEM) was used to observe the membrane surface information before and after treatment.

The global separation performance of a membrane process is typically defined by water flux and salt rejection.

Water flux *J* (L/m^2^h) was calculated according to Equation (1):

(1) $$J=\frac{\Delta v}{A\Delta t}$$

∆*v*—volume of permeate collected over ∆*t* period, L.

*A*—membrane effective separation area, m^2^.

∆*t*—time of sample collection, h.

Salt rejection R (%) was calculated according to Equation (2):

(2) $$R=\frac{C_{f}-C_{P}}{C_{f}}\times 100\%$$

*C_f_*—influent conductivity, μs/cm.

*C_P_*—permeate conductivity, μs/cm.

## 3. Results and Discussion

### 3.1. Water Quality Analysis

By comparing the indicators of chloride ion, total iron, pH, total alkalinity, and total hardness in the wastewater quality with the corresponding standard limits, the test results of chloride ion and total iron were obtained to be lower than the standard limits (Table 5). However, the test results for pH, total alkalinity, and total hardness exceeded the standard limits, indicating that these indicators did not meet the water quality requirements. By identifying the indicators that do not meet the standard, the object of concern was further clarified, and appropriate measures were taken to deal with them.

### 3.2. Comparison and Selection of Nanofiltration Membranes

At present, NF/RO is widely used in the advanced treatment of wastewater [26,27]. NF/RO has excellent removal efficiency and selectivity. Here, we selected three commercial nanofiltration membranes (NF90, NF270, XN45) and one reverse osmosis membrane (XLE) for membrane filtration tests.

Comparing the treatment results of the four membranes (Table 6), NF90 and XLE demonstrated superior removal efficiencies for each pair of contaminants compared to the other two nanofiltration membranes. It was reported that charge repulsion plays a major role in the repulsion mechanism of NF/RO membranes to ionic solutes. The active layer pore size of the NF90 membrane is smaller, ranging from 0.34 nm to 0.81 nm, while the NF270 membrane and XN45 membrane pore size are slightly larger, ranging from 0.38 nm to 1.56 nm. The pores in the NF membrane are crucial to its ability to repel ionic solutes efficiently [28]. As a reverse osmosis membrane, XLE has excellent performance in separating small monomer ions through particle size screening and the Donnan exclusion mechanism [29]. However, the pH value of the effluent treated by the XLE membrane is only 3.64, which may be due to the surface charge characteristics of the membrane leading to changes in the concentration of specific ions in the effluent water, thus affecting the overall pH value [12].

**Figure 1:**
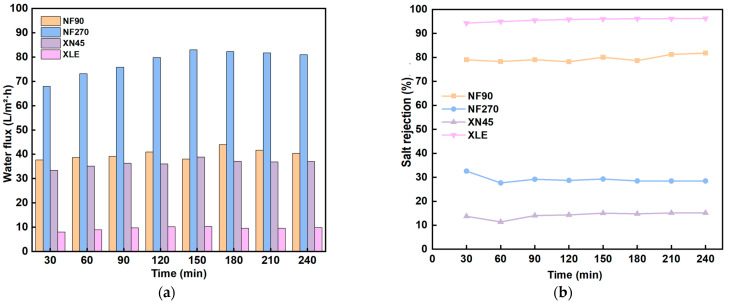
Water flux (**a**) and salt rejection (**b**) of nanofiltration membranes at different operating times.

Figure 1 shows that the NF90 membrane achieves a stable salt rejection of approximately 80%, indicating its effectiveness in retaining most salts present in the water. In nanofiltration systems, water flux (Figure 1a) and salt rejection (Figure 1b) are critical metrics for evaluating performance. The 80% salt rejection indicates that the NF90 has high efficiency in treating salty water. However, for XN45 and XLE membranes, there is a lower level of water flux. The NF270 membrane exhibits the highest water flux, but it has a lower salt rejection, resulting in a conductivity of 2171 μs/cm. This indicates that the NF270 is less effective than the NF90 in removing salts.

In summary, both salt rejection and the water flux of nanofiltration membranes must be considered. Although NF270 has high water flux, its low salt rejection makes the effluent quality poor. Conversely, while the water flux of the NF90 is slightly lower than that of the NF270, its stable salt rejection of approximately 80% ensures that the effluent quality meets high standards. Therefore, after evaluating both water flux and salt rejection, the NF90 was selected for the subsequent experiment.

### 3.3. Determination of Optimum Operating Parameters for NF90

**Figure 2:**
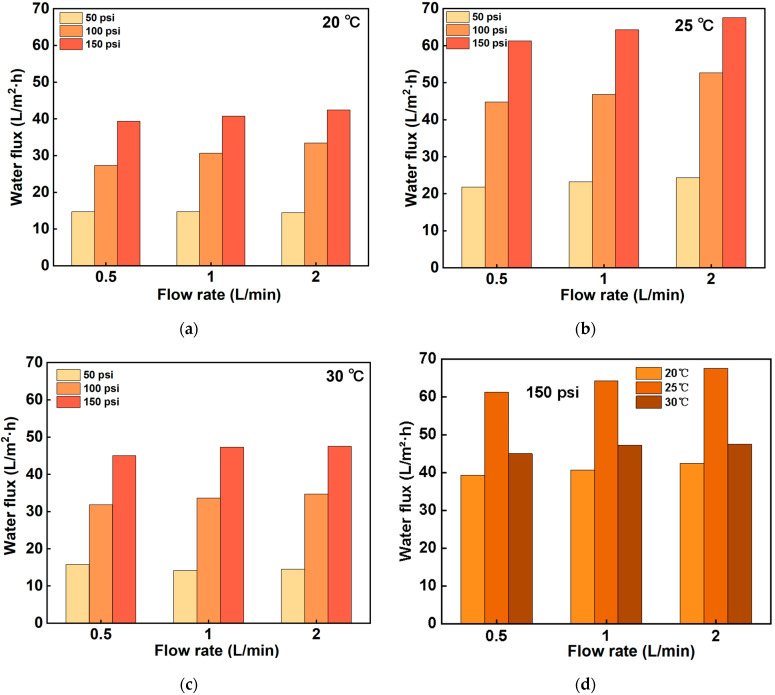
Average water flux based on the NF90 at (**a**) 20 °C, (**b**) 25 °C, and (**c**) 30 °C for different flow rates and pressures. (**d**) Average water flux of NF90 at 150 psi at different temperatures and flow rates.

Refer to the Dow Membrane Technical Manual (2022) for the operating parameters of NF90, as shown in Table 7. Figure 2 shows the NF90 average water flux and average salt rejection. The result shows that the water flux of the NF90 membrane is positively correlated with the increase in pressure. This phenomenon was consistent across all three temperature conditions, suggesting that the NF90 membrane exhibits similar response characteristics to variations in pressure and the flow rate at different temperatures. Furthermore, the average salt rejection reached its maximum at all temperature conditions when the pressure was set to 150 psi. This finding implies that, at higher operating pressures, the NF90 membrane effectively retains salts, thereby enhancing wastewater treatment efficiency.

At the same time, it can be observed that the maximum water flux occurs at 25 °C, and the water flux tends to decrease after the temperature rises. Temperature plays an important role in changing the membrane’s structure and the physical properties of the solution. Due to the expansion of pore size, the retention behavior of all membranes changes. Moreover, the transmembrane pressure (TMP) of the membranes will decrease at higher temperatures, so that only a small water flux can be obtained. Therefore, the maximum water flux can be achieved at the right temperature [30].

In addition, the experiment also showed that the average water flux of the NF90 reached the maximum value of 69.52 L/m^2^·h at 25 °C and a flow rate of 2 L/min. This result suggests that the NF90 can perform optimally in terms of water flux at moderate temperatures and high flow rates.

**Figure 3:**
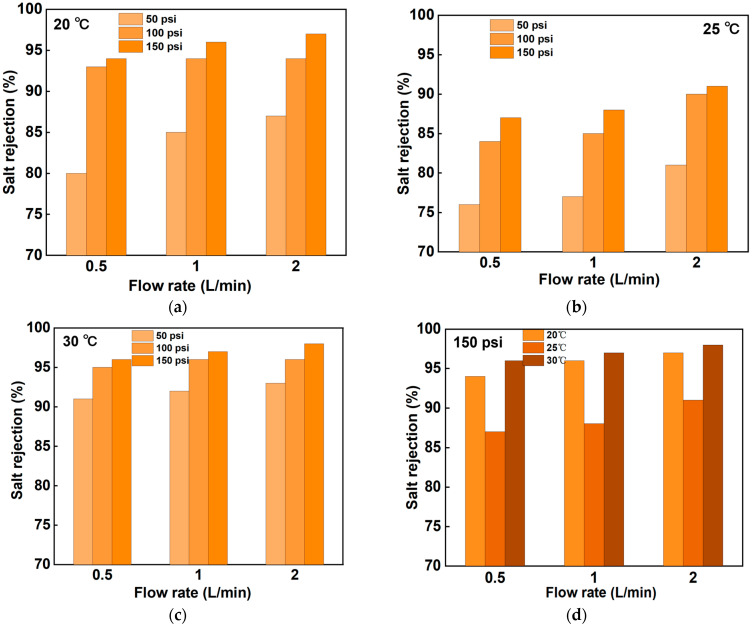
Average NF90 salt rejection at different flow rates and pressures: (**a**) 20 °C, (**b**) 25 °C, and (**c**) 30 °C. (**d**) Average salt rejection corresponding to different temperatures and flow rates at 150 psi.

According to the results presented in Figure 3, the average salt rejection under different conditions demonstrates a general trend of decreasing and then increasing with rising temperatures, with the highest salt rejection observed at 30 °C. This observation reflects the complex physical and chemical phenomena involved in membrane separation. Previous studies on the effect of temperature on scaling have shown that reducing the temperature from 22 °C to 15 °C increases concentration polarization due to the high viscosity of the solution. At the same time, the diffusion coefficient decreases at lower temperatures, resulting in the lower permeability of water and solute through the membrane [31,32,33]. At low temperatures, membrane permeability is low, resulting in high salt retention. With the increase in temperature, the permeability of the membrane increases, resulting in a decrease in the desalting rate. However, with further temperature increases, changes in membrane material properties or the ionization of salts may enhance retention, leading to subsequent increases in salt removal rates.

From Figure 3a–c, it is evident that salt rejection reached its highest value at all pressure settings of 150 psi, and this result was consistent across different temperatures. Furthermore, Figure 3d illustrates that the average salt rejection can be as high as 90% under the same pressure across varying temperatures, highlighting the exceptional salt retention performance of the NF90 nanofiltration membrane in a high-pressure environment. Notably, at 30 °C and a flow rate of 2 L/min, the average salt rejection can even reach 98%, further confirming the effectiveness of high-pressure conditions in enhancing the salt rejection capabilities of NF90 membranes.

However, in practical applications, factors such as energy consumption, operating costs, and the equipment lifespan must be considered. While higher pressure and flow rates may yield greater average salt rejection, they can also lead to increased energy consumption and accelerated wear of the equipment. Therefore, after evaluating these various factors, 25 °C, 150 psi, and 1 L/min were determined to be the optimal operating conditions for the NF90 membrane. This combination effectively balances a high salt rejection with controlled energy consumption and operating costs, demonstrating significant practical value.

Based on these optimal operating conditions, the next step of this experimental study was conducted to further verify the performance of the NF90 membrane and to provide support for its implementation in practical applications.

### 3.4. Continuous Operation Results

Nanofiltration membrane fouling is inevitable, resulting in increased differential pressure across the membrane, reduced flux, and shortened membrane lifespan. This phenomenon adversely affects process stability and economic efficiency [34].

**Figure 4:**
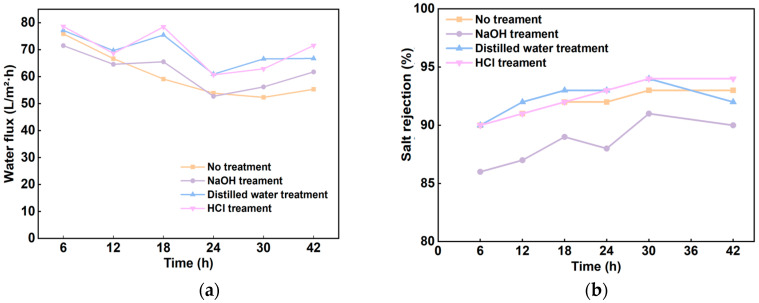
Water flux (**a**) and salt rejection (**b**) at different operating times after treating membrane fouling with different methods.

**Figure 5:**
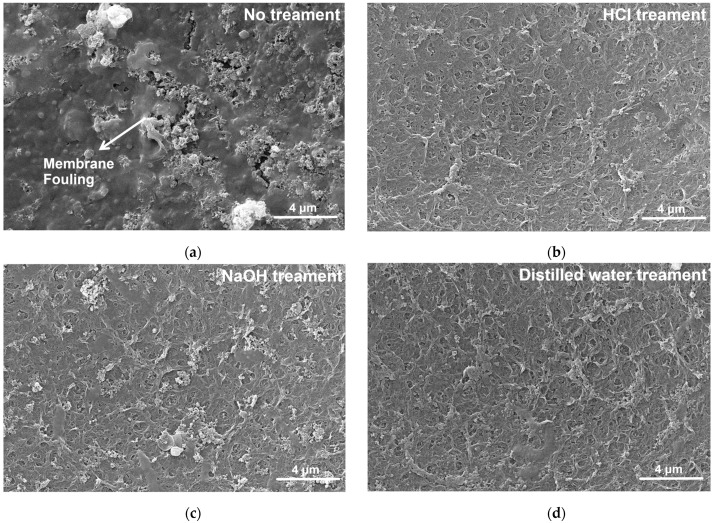
SEM images of NF90 after treatment with different methods (**a**) no treatment, (**b**) HCl treatment, (**c**) NaOH treatment, and (**d**) distilled water treatment.

The water flux decreased significantly after 12 h of continuous operation under the optimal experimental parameters (Figure 4a). Following treatment through various methods, the water flux exhibited a substantial increase, indicating an enhancement in membrane performance, particularly after HCl treatment. Compared with the untreated condition, the salt rejection rate of the membranes was improved after treatment (Figure 4a). It has been reported that acid cleaning can make the membrane tighter through charge neutralization and thus play a role in maintaining the membrane’s ion retention ability [35]. As illustrated by the SEM images of the membranes treated by each method in Figure 5, the images for HCl treatment (Figure 5b) and clear water treatment (Figure 5d) displayed fewer impurities compared to the other treatments. Both methods achieved a salt rejection of 90% during the later stages of operation. Pore expansion occurs in the PA NF membranes after alkaline cleaning (pH = 11), and small dirt enters the expanded pores and remains tightly in the membrane, resulting in more serious dirt and delaying the release of dirt during cleaning. Therefore, the effect of alkaline cleaning in practical applications is getting worse and worse [36].

The membranes were cleaned with water and HCl every 6 h over 48 h in the subsequent experiments. The data presented in Table 8 show that the water quality parameters significantly improved following both NF90 and HCl treatments, with the pH value remaining within a moderate range. Overall, the treatment process led to a notable enhancement in water quality.

The comparison indicated that the water quality of the membrane effluent after the treatment with an HCl solution (with a pH value between 3 and 4) was superior to that of the clear water treatment. Specifically, the conductivity was reduced to 2350.3 μs/cm, and the total hardness was measured at 17.02 mg/L, with other parameters also meeting regulatory standards by the water quality of urban wastewater recycling for industrial use (GB/T 19923-2024 [20]). This finding confirms the effectiveness of HCl treatment and provides valuable data support for subsequent water treatment processes.

## 4. Conclusions

In this study, an evaluation of the performance of various nanofiltration membranes revealed that the NF90 membrane exhibited superior efficacy in treating wastewater samples from a specific enterprise. Through the precise regulation of experimental conditions, it was determined that the NF90 achieved its optimal operating conditions at a temperature of 25 °C, an operating pressure of 150 psi, and a flow rate of 1 L/min. This configuration not only ensured excellent water flux but also achieved a very high salt rejection, providing a solid foundation for parameter optimization in practical applications. To address the issue of membrane fouling that may arise from long-term operation, a long-term operational test was conducted. A maintenance strategy involving regular cleaning with an acidic solution at pH 3–4 effectively removed contaminants adhering to the membrane surface and mitigated the impact of fouling. The implementation of high-performance nanofiltration membrane technology not only meets the urgent needs of corporate environmental management but also significantly enhances wastewater treatment efficiency. Furthermore, it serves as a valuable reference for similar wastewater treatment projects and engineering practices.

## Figures and Tables

**Table 1:** Membrane properties.

Membrane	Type	Water Flux (L/m^2^·h)	Interception Molecular Weight (Dalton)	Rejection Rate (%)
NF90	NF	43	100–150	≥99.0
NF270	NF	70	300–400	≥97.0
XN45	NF	60	400–700	90.0–97.0
XLE	RO	30	200–300	≥99

**Table 2:** Wastewater source and proportion of each part.

Source of Industrial Wastewater	Proportion (%)
storage tank	11.54%
quality control workshop	5.77%
adhesive coating workshop	5.77%
soft water daily tank	76.92%

**Table 3:** Water quality parameters of wastewater.

**Water Quality Parameters**	**Conductivity (μs/cm)**	**pH**	**Total** **Alkalinity (mg/L** **)**	**Total Hardness (mg/L** **)**	**Cl^−^ (mg/L)**	**Total Iron (mg/L)**
result	2958	8.81	380	810	200	0.05

**Table 4:** The national standard for industrial water reuse in China (GB/T 19923-2024 [20]) for circulating cooling water quality standards.

Item	Maximum Contaminant Level
pH	6.5~8.5
BOD (mg/L)	≤10
COD (mg/L)	≤60
Cl^−^ (mg/L)	≤250
total hardness	≤450
total alkalinity	≤350
TP (mg/L)	≤1

**Table 5:** Comparison of wastewater quality index and China’s national standard for industrial water reuse (GB/T 19923-2024 [20]).

Item	Result	Maximum Contaminant Level	Unit
Cl^−^	200	≤250	mg/L
total iron	0.05	≤0.3	mg/L
total alkalinity	380	≤350	mg/L
total hardness	810	≤450	mg/L
pH	8.81	6.0~9.0	

**Table 6:** Water quality parameters of wastewater treated with four types of nanofiltration membranes.

Water Quality Parameters	Wastewater	NF90	NF270	XN45	XLE
COD	52	<1	23	14	<1
pH	7.71	5.50	3.29	6.93	3.64
conductivity (μs/cm)	3035	553.6	2171	2576	114.1
total alkalinity (mg/L)	237.7	145.15	200.2	187.69	150.15
total chlorine (mg/L)	1.6	0.63	1.12	0.63	1.12

**Table 7:** Operating parameters of NF90 in Dow Membrane Technical Manual (2022).

Operating Parameters	Pressure (psi)	Flow Rate (L/min)	Temperature (°C)
NF90	70	1.8	<45 °C

**Table 8:** Comparison of wastewater quality indices with the quality of wastewater treated with NF90.

Method of Treatment	Conductivity (μs/cm)	pH	Total Alkalinity (mg/L)	Total Hardness (mg/L)	Cl^−^(mg/L)	Total Iron (ug/L)	COD (mg/L)
wastewater	3096	7.92	50.69	326.33	9.65	9.57	52
NF90 treated with distilled water	292.8	6.62	38.79	28.03	8.70	2.78	19
NF90 treated with HCl	235.3	6.86	23.77	17.02	8.42	2.05	15

## Data Availability

The original contributions presented in this study are included in the article/Supplementary Material.

## Supplementary Materials

The following supporting information can be downloaded at: https://www.mdpi.com/article/10.3390/membranes14120266/s1.
