# Supplementary material for: A Case Study on Recycling Industrial Wastewater with Nanofiltration Membrane Separation Technology
Source: Membranes (Basel). 2024 Dec 9;14(12):266. doi: 10.3390/membranes14120266 (PMC11676913; doi:10.3390/membranes14120266)
Supplement: Supplementary file 1 [file membranes-14-00266-s001.zip › membranes-3294647-supplementary.pdf]

Supplementary Material

A case study on recycling industrial wastewater with nanofiltration membrane separation technology

Yiqiang Deng[1,2], Xiaoqian Bai[1,2], Jialong Lin[1,2], Linyan Yang[1,2], Yuqing Lin[1,2], Mingyue Lin[1,2,3,*], Guangli Xiu[1,2,*]

[1]State Environmental Protection Key Laboratory of Environmental Risk Assessment and Control on Chemical Process, East China University of Science and Technology, Shanghai 200237, P.R. China;

[2]Shanghai Environmental Protection Key Laboratory on Environmental Standard and Risk Management of Chemical Pollutants, School of Resources and Environmental Engineering, East China University of Science and Technology, Shanghai 200237, P.R. China;

[3]Shanghai Institute of Pollution Control and Ecological Security, Shanghai 200092, P.R. China

[*]Correspondence: Yifeng L.: luyifeng@scip.gov.cn; Mingyue L.: ms.mingyuelin@ecust.edu.cn; Guangli X.: xiugl@ecust.edu.cn;

Table S1. Experimental data of optimal membrane selection.

		NF90	NF270	XLE	XN45
30 min	Water flux(L/m^2·h)	45.71	83.29	7.86	29.29
	Salt rejection	85.56%	30.44%	95.14%	16.57%
60 min	Water flux(L/m^2·h)	45.86	86.57	9.14	30.29
	Salt rejection	85.83%	29.66%	95.61%	15.70%
90 min	Water flux(L/m^2·h)	46.29	89.71	9.29	30.71
	Salt rejection	86.32%	29.91%	95.42%	16.31%
120 min	Water flux(L/m^2·h)	45.86	90.43	9.43	31.00
	Salt rejection	86.83%	29.76%	96.01%	16.22%
150 min	Water flux(L/m^2·h)	45.29	90.14	9.43	31.14
	Salt rejection	87.21%	29.18%	96.06%	16.04%
180 min	Water flux(L/m^2·h)	44.57	89.00	9.86	31.29
	Salt rejection	87.52%	29.25%	96.12%	16.33%
210 min	Water flux(L/m^2·h)	45.29	98.57	10.57	32.29
	Salt rejection	87.75%	29.02%	96.15%	16.22%
240 min	Water flux(L/m^2·h)	43.43	95.57	9.71	31.43
	Salt rejection	87.99%	28.84%	96.18%	16.28%

Table S2. Repeated experimental data of optimal membrane selection.

		NF90	NF270	XLE	XN45
30 min	Water flux(L/m^2·h)	37.71	68.00	8.00	33.43
	Salt rejection	78.99%	32.57%	94.30%	13.74%
60 min	Water flux(L/m^2·h)	38.71	73.14	8.86	35.14
	Salt rejection	78.28%	27.65%	94.95%	11.39%
90 min	Water flux(L/m^2·h)	39.14	75.86	9.71	36.29
	Salt rejection	79.04%	29.18%	95.49%	14.04%
120 min	Water flux(L/m^2·h)	41.00	79.86	10.14	36.00

	Salt rejection	78.19%	28.70%	95.81%	14.32%
150 min	Water flux($L/m^2 \cdot h$)	38.00	83.00	10.29	38.86
	Salt rejection	80.06%	29.29%	96.02%	15.03%
180 min	Water flux($L/m^2 \cdot h$)	44.00	82.29	9.57	37.14
	Salt rejection	78.66%	28.49%	96.14%	14.77%
210 min	Water flux($L/m^2 \cdot h$)	41.71	81.71	9.57	36.86
	Salt rejection	81.26%	28.45%	96.18%	15.13%
240 min	Water flux($L/m^2 \cdot h$)	40.43	81.00	9.86	37.00
	Salt rejection	81.76%	28.47%	96.24%	15.12%

Table S3. Experimental data of optimal parameters determined by orthogonal test.

Operating condition	NF	Water flux ($L/m^2 \cdot h$)	Salt rejection	Average water flux ($L/m^2 \cdot h$)	Water flux standard deviation	Average salt rejection	Average salt rejection standard deviation
20 °C 50 psi 0.5 L/min	1-NF90	15.57	81%	14.75	0.96	80%	0.03
	2-NF90	13.71	77%				
	3-NF90	14.14	80%				
	4-NF90	15.57	84%				
20 °C 50 psi 1 L/min	1-NF90	15.86	86%	14.71	1.02	85%	0.03
	2-NF90	13.71	81%				
	3-NF90	14.00	84%				
	4-NF90	15.29	87%				
20 °C 50 psi 2 L/min	1-NF90	15.86	89%	14.46	1.38	87%	0.02
	2-NF90	13.14	84%				
	3-NF90	13.43	87%				
	4-NF90	15.43	90%				
20 °C 100 psi	1-NF90	28.27	93%	27.35	1.76	93%	0.008
	2-NF90	25.14	92%				
	3-NF90	26.71	93%				

0.5 L/min	4-NF90	29.14	94%				
20 ℃<br>100 psi<br>1 L/min	1-NF90	32.00	94%	30.68	2.19	94%	0.008
	2-NF90	28.00	93%				
	3-NF90	29.86	94%				
	4-NF90	32.86	95%				
20 ℃<br>100 psi<br>2 L/min	1-NF90	35.29	94%	33.47	2.91	94%	0.0125
	2-NF90	29.86	92%				
	3-NF90	32.43	94%				
	4-NF90	36.29	95%				
20 ℃<br>150 psi<br>0.5 L/min	1-NF90	40.29	95%	39.37	2.3	95%	0.05
	2-NF90	36.49	94%				
	3-NF90	38.71	95%				
	4-NF90	42.00	95%				
20 ℃<br>150 psi<br>1 L/min	1-NF90	41.57	96%	40.75	2.9	96%	0
	2-NF90	37.14	96%				
	3-NF90	40.14	96%				
	4-NF90	44.14	96%				
20 ℃<br>150 psi<br>2 L/min	1-NF90	44.00	97%	42.47	0.97	97%	0.005
	2-NF90	38.29	96%				
	3-NF90	41.29	97%				
	4-NF90	46.29	97%				
25 ℃<br>50 psi<br>0.5 L/min	1-NF90	23.19	79%	21.79	1.09	76%	0.04
	2-NF90	21.44	78%				
	3-NF90	21.96	75%				
	4-NF90	20.59	70%				
25 ℃<br>50 psi<br>1 L/min	1-NF90	25.76	79%	23.21	1.78	77%	0.04
	2-NF90	22.81	80%				
	3-NF90	21.66	76%				
	4-NF90	22.60	71%				

25 °C 50 psi 2 L/min	1-NF90	24.38	81%	24.34	0.35	81%	0.018
	2-NF90	24.30	81%				
	3-NF90	24.59	82%				
	4-NF90	24.09	80%				
25 °C 100 psi 0.5 L/min	1-NF90	48.70	87%	44.74	2.9	84%	0.029
	2-NF90	44.04	86%				
	3-NF90	41.57	84%				
	4-NF90	44.66	80%				
25 °C 100 psi 1 L/min	1-NF90	52.87	87%	46.83	5.7	85%	0.029
	2-NF90	49.09	87%				
	3-NF90	45.94	85%				
	4-NF90	39.41	81%				
25 °C 100 psi 2 L/min	1-NF90	52.66	90%	52.66	0.36	90%	0.0098
	2-NF90	52.53	92%				
	3-NF90	51.90	91%				
	4-NF90	49.55	87%				
25 °C 150 psi 0.5 L/min	1-NF90	65.74	88%	61.25	3.09	87%	0.018
	2-NF90	59.53	88%				
	3-NF90	58.94	88%				
	4-NF90	60.80	84%				
25 °C 150 psi 1 L/min	1-NF90	67.86	90%	64.29	2.65	88%	0.024
	2-NF90	64.74	90%				
	3-NF90	62.29	88%				
	4-NF90	62.28	85%				
25 °C 150 psi 2 L/min	1-NF90	25.08	92%	67.56	2.77	91%	0.23
	2-NF90	69.53	93%				
	3-NF90	67.6	91%				
	4-NF90	65.6	89%				
	1-NF90	15.71	91%	15.79	1.68	91%	0.0094

30 °C 50 psi 0.5 L/min	2-NF90	14.29	90%				
	3-NF90	15.00	91%				
	4-NF90	18.14	92%				
230 °C 50 psi 1 L/min	1-NF90	14.00	92%	14.14	0.89	92%	0.0097
	2-NF90	13.43	91%				
	3-NF90	13.71	92%				
	4-NF90	15.43	93%				
30 °C 50 psi 2 L/min	1-NF90	14.57	93%	14.50	0.99	93%	0.0093
	2-NF90	13.57	92%				
	3-NF90	14.00	93%				
	4-NF90	15.86	94%				
30 °C 100 psi 0.5 L/min	1-NF90	28.14	94%	31.82	2.90	95%	0.0062
	2-NF90	33.43	94%				
	3-NF90	31.00	95%				
	4-NF90	34.71	96%				
30 °C 100 psi 1 L/min	1-NF90	34.57	96%	33.64	2.55	96%	0.0053
	2-NF90	30.57	95%				
	3-NF90	32.86	96%				
	4-NF90	36.58	96%				
30 °C 100 psi 2 L/min	1-NF90	35.86	96%	34.71	2.84	96%	0.0047
	2-NF90	31.43	96%				
	3-NF90	33.57	97%				
	4-NF90	38.00	97%				
30 °C 150 psi 0.5 L/min	1-NF90	44.86	96%	45.00	3.12	96%	0.0034
	2-NF90	41.58	95%				
	3-NF90	44.483	96%				
	4-NF90	49.14	96%				
30 °C	1-NF90	47.71	97%	47.32	3.87	97%	0.0035
	2-NF90	42.71	97%				

150 psi<br><br>1 L/min	3-NF90	46.71	97%				
	4-NF90	52.14	97%				
30 °C<br><br>150 psi<br><br>2 L/min	1-NF90	48.71	98%	47.57	3.08	98%	0.0029
	2-NF90	43.43	97%				
	3-NF90	50.71	98%				
	4-NF90	47.43	98%				

Table S4. Different methods were used to process the experimental data of membrane contamination.

Running time	treatment method	Water flux ($L/m^2 \cdot h$)	Salt rejection	Average water flux ($L/m^2 \cdot h$)	Average salt rejection
6 h	No treatment	71.49	86%	75.76	89%
		77.14	90%		
		75.87	90%		
		78.60	90%		
12 h	No treatment	64.57	87%	67.38	90%
		69.63	92%		
		66.67	91%		
		68.63	91%		
18 h	NaOH treatment	65.50	89%	69.62	92%
	Distilled water treatment	75.44	93%		
	No treatment	59.10	92%		
	HCl treatment	78.43	92%		
24 h	NaOH treatment	44.86	96%	52.70	88%

	Distilled water treatment	41.57	95%		
	No treatment	44.43	96%		
	HCl treatment	49.14	96%		
30 h	NaOH treatment	56.177	91%	59.48	93%
	Distilled water treatment	66.57	94%		
	No treatment	52.30	93%		
	HCl treatment	62.89	94%		
42 h	NaOH treatment	61.74	90%	63.83	92%
	Distilled water treatment	66.74	92%		
	No treatment	55.29	93%		
	HCl treatment	71.54	94%		
